# Acquisition of epithelial-mesenchymal transition phenotype in the tamoxifen-resistant breast cancer cell: a new role for G protein-coupled estrogen receptor in mediating tamoxifen resistance through cancer-associated fibroblast-derived fibronectin and β1-integrin signaling pathway in tumor cells

**DOI:** 10.1186/s13058-015-0579-y

**Published:** 2015-05-21

**Authors:** Jie Yuan, Manran Liu, Li Yang, Gang Tu, Qing Zhu, Maoshan Chen, Hong Cheng, Haojun Luo, Weijie Fu, Zhenhua Li, Guanglun Yang

**Affiliations:** Department of Endocrine and Breast Surgery, the First Affiliated Hospital of Chongqing Medical University, #1 You-Yi Rd, Yu-zhong District, Chongqing, 400016 China; Key Laboratory of Laboratory Medical Diagnostics, Chinese Ministry of Education, Chongqing Medical University, Chongqing, 400016 China

## Abstract

**Introduction:**

Acquired tamoxifen resistance remains the major obstacle to breast cancer endocrine therapy. β1-integrin was identified as one of the target genes of G protein-coupled estrogen receptor (GPER), a novel estrogen receptor recognized as an initiator of tamoxifen resistance. Here, we investigated the role of β1-integrin in GPER-mediated tamoxifen resistance in breast cancer.

**Methods:**

The expression of β1-integrin and biomarkers of epithelial-mesenchymal transition were evaluated immunohistochemically in 53 specimens of metastases and paired primary tumors. The function of β1-integrin was investigated in tamoxifen-resistant (MCF-7R) subclones, derived from parental MCF-7 cells, and MCF-7R β1-integrin-silenced subclones in MTT and Transwell assays. Involved signaling pathways were identified using specific inhibitors and Western blotting analysis.

**Results:**

GPER, β1-integrin and mesenchymal biomarkers (vimentin and fibronectin) expression in metastases increased compared to the corresponding primary tumors; a close expression pattern of β1-integrin and GPER were in metastases. Increased β1-integrin expression was also confirmed in MCF-7R cells compared with MCF-7 cells. This upregulation of β1-integrin was induced by agonists of GPER and blocked by both antagonist and knockdown of it in MCF-7R cells. Moreover, the epidermal growth factor receptor/extracellular regulated protein kinase (EGFR/ERK) signaling pathway was involved in this transcriptional regulation since specific inhibitors of these kinases also reduced the GPER-induced upregulation of β1-integrin. Interestingly, silencing of β1-integrin partially rescued the sensitivity of MCF-7R cells to tamoxifen and the α5β1-integrin subunit is probably responsible for this phenomenon. Importantly, the cell migration and epithelial-mesenchymal transition induced by cancer-associated fibroblasts, or the product of cancer-associated fibroblasts, fibronectin, were reduced by knockdown of β1-integrin in MCF-7R cells. In addition, the downstream kinases of β1-integrin including focal adhesion kinase, Src and AKT were activated in MCF-7R cells and may be involved in the interaction between cancer cells and cancer-associated fibroblasts.

**Conclusions:**

GPER/EGFR/ERK signaling upregulates β1-integrin expression and activates downstream kinases, which contributes to cancer-associated fibroblast-induced cell migration and epithelial-mesenchymal transition, in MCF-7R cells. GPER probably contributes to tamoxifen resistance via interaction with the tumor microenvironment in a β1-integrin-dependent pattern. Thus, β1-integrin may be a potential target to improve anti-hormone therapy responses in breast cancer patients.

**Electronic supplementary material:**

The online version of this article (doi:10.1186/s13058-015-0579-y) contains supplementary material, which is available to authorized users.

## Introduction

Tamoxifen, a selective estrogen receptor (ER) modulator, is the most frequently used anti-hormonal drug for the adjuvant treatment of women with ER-positive breast cancer [1]. Acquired resistance is still the major clinical challenge to the therapeutic efficacy of tamoxifen. A growing amount of evidence has demonstrated that the aberrant activated growth factor signaling pathways contribute to tamoxifen resistance [2, 3]. However, most studies [4, 5] have tested the hypothesis that tamoxifen resistance results from genetic alterations and autocrine or paracrine mechanisms in the epithelial tumor cells themselves. Tumors are complex organs comprising a variety of components such as tumor cells, fibroblasts, immune cells, vessels, and extracellular matrix. The role of the tumor microenvironment in tumor progression and drug resistance is gradually being clarified [6, 7].

One of the crucial reasons for drug resistance is the metastasis of cancer cells to secondary sites [8, 9]. Tumor cells achieve this by activating an epithelial-mesenchymal transition (EMT) program to experience phenotypic alterations, such as the loss of cell-cell interactions and the gain of cell mobility to evade from the primary lesion. Molecular hallmarks of EMT include the loss of epithelial markers, such as E-cadherin, the gain of the expression of mesenchymal markers, such as N-cadherin, vimentin and fibronectin, the loss of cell polarity, and reorganization of the actin cytoskeleton accompanied by the morphological change [10, 11]. For example, tamoxifen-resistant MCF-7 breast cancer cells (MCF-7R) display enhanced motile and invasive behavior as well as accompanying EMT-like properties compared to the parental MCF-7 cell line *in vitro* [12, 13]. Emerging evidence suggests a close association between drug resistance and the induction of EMT in cancer [10, 14]; however, the initiator and the specific mechanism of EMT during the development of tamoxifen resistance remain to be determined.

G protein-coupled estrogen receptor (GPER), also called G protein-coupled receptor 30 (GPR30), is a novel ER that can be activated by tamoxifen and the pure anti-estrogen fulvestrant. This receptor has been shown to be important in the induction of tamoxifen resistance through the GPER/epidermal growth factor receptor (EGFR) signaling pathway [15, 16]. Moreover, it was demonstrated GPER functions as an important initiator in the development of tamoxifen resistance in hormone-dependent breast cancer [17]. In order to further disclose the potential role of GPER in the tamoxifen-resistant ER^+^ breast cancer, we identified a set of target genes in MCF-7R subclones using cDNA microarray (data unpublished). One of these genes, β1-integrin, has been demonstrated to play a key role in tumor progression and tumor survival [18, 19]. Furthermore, β1-integrin, which coordinates much broader functional processes such as inflammation, proliferation, adhesion, and invasion, has recently been implicated in therapeutic resistance in multiple solid cancer models [20, 21]. Importantly, integrins mediate signal transduction between the tumor cell and its microenvironment, which complicates the identification of the mechanisms underlying drug resistance. Few studies have reported the involvement of β1-integrin in tamoxifen resistance. Pontiggia and colleagues [22] demonstrated that soluble factors or extracellular matrix components in the microenvironment protect against tamoxifen-induced cell death through interaction with β1-integrin using tamoxifen-sensitive breast cancer cells. However, this finding does not clarify the role of β1-integrin in resistant cells due to the diversity of the cell model.

In this study, we elucidated that the interaction of the cancer-associated fibroblast (CAF)-derived tumor microenvironment and β1-integrin locating on the cell membrane of MCF-7R cells plays a key role in GPER-mediated tamoxifen resistance. Increased β1-integrin was detected in metastasis (MT) breast carcinoma specimens compared with their matched primary tumors (PTs). Knockdown of β1-integrin expression in MCF-7R cells partly restored tamoxifen sensitivity and suppressed the EMT phenotype facilitated by CAFs, or the product of CAFs, fibronectin, *in vitro*. These findings suggest that targeting β1-integrin may be a promising solution to tamoxifen resistance in breast cancer.

## Methods

### Materials

The rabbit monoclonal (EP1041Y) antibody against β1-integrin was obtained from Abcam (Cambridge, UK). Antibodies against Src (pY418 and total), focal adhesion kinase (FAK; pY397 and total), extracellular-signal regulated kinase-1 and -2 (ERK1/2; pT202/Y204), E-cadherin, vimentin and fibronectin were purchased from Bioworld (Saint Louis Park, MN, USA). Antibodies against AKT (pS473 and total) were from Cell Signaling Technology (New England Biolabs, Herts, UK). β1-integrin antisense oligonucleotides and GAPDH antisense oligonucleotides were purchase from Invitrogen (New York, USA). Fibronectin, 4-hydroxytamoxifen (TAM) and (4,5-dimethylthiazol-2-yl)-2,5-diphenyltetrazolium bromide (MTT) were obtained from Sigma-Aldrich (Steinheim, Germany). GPER agonist G1 and antagonist G15 were purchased from Tocris (Missouri, USA). Function-blocking integrin monoclonal antibodies specific for α2β1-integrins (AK-7) and α5β1 integrin (P1D6) were from EMD Biosciences (San Diego, CA, USA). All other reagents were from Beyotime (Haimen, China).

### Cell culture

Human MCF-7 breast carcinoma cells (MCF-7), purchased from the Institute of Biochemistry and Cell Biology, Chinese Academy of Sciences (IBCB, Shanghai, China), were routinely grown in Dulbecco's modified Eagle's medium (DMEM; Gibco, Rockville, MD, USA) containing 5% fetal bovine serum (FBS; Gibco), 10 μg/ml insulin, 100 IU/ml penicillin and 100 μg/ml streptomycin. The tamoxifen-resistant sublines (MCF-7R) [23] were derived from MCF-7 by continuous exposure to TAM diluted in 0.1% ethanol. MCF-7R cells were cultured continuously in medium containing 5% FBS supplemented with 100 nM TAM. Before all experiments, cells were switched to phenol-free DMEM containing 0.5% charcoal-dextran-stripped FBS for 2 days, except where noted.

MDA-MB-468, MDA-MB-231, and SKBR3 cells were kindly provided by Dr Gang Tu, and the culture conditions were as described previously [24].

CAFs, a gift from Dr Manran Liu, were isolated and cultured as described previously [25].

No consent was needed from any patients or Institute Ethics Board, because only commercially available cell lines were used in this study.

### Specimens

The 77 paired archival paraffin-embedded breast cancer specimens were obtained from the Clinical Diagnostic Pathology Center, Chongqing Medical University (Chongqing, China). All patient details and exclusion criteria have been described previously [17]. All patients received tamoxifen only after surgery. All patients involved in this study consented to participate in the study and publication of its results. The experiments were approved by the Ethics Committee of the First Affiliated Hospital of Chongqing Medical University and were conducted in accordance with the Helsinki Declaration.

### Immunohistochemistry

Sections of formalin-fixed and paraffin-embedded breast cancer specimens or corresponding recurrent lesions were mounted on SuperFrost Plus glass slides (ZsBio, Beijing, China) and dried overnight. The immunohistochemical protocols are described elsewhere [17]. The slides were incubated with affinity-purified rabbit anti-β1-integrin (1:100), anti-GPER (1:250), anti-E-cadherin (1:200), anti-vimentin (1:200) and anti-fibronectin (1:200) for 2 hours at 37 °C.

### Evaluation of β1-integrin and G protein-coupled estrogen receptor staining

The β1-integrin expression in samples was scored based on intensity (0 to 3) and extent (0 = <10%, 1 = 10 to 25%, 2 = 26 to 50%, 3 = >50%) ato ccording previously described criteria [26]. The individual categories were multiplied to give a total immunohistochemical score ranging between 0 and 9. Samples that scored ≥3 were defined as positive immunohistochemical results.

GPER expression was classified as described previously [17] and scores were assigned as follows: the percentage of positive cells was categorized as 0 (negative staining in all cells), 1 (<1% cells stained), 2 (1 to 10% of cells stained), 3 (11 to 40% cells stained), 4 (41 to 70% cells stained) or 5 (71 to 100% cells stained), and staining intensity was categorized as 0 (negative), 1 (weak), 2 (moderate) or 3 (strong). Percentage and intensity scores were added to give total immunohistochemical scores, ranging from 0 to 8. Specimens that scored ≥2 were defined as GPER^+^. In our previous work, 53 recurrent breast cancer specimens were identified as GPER-positive and the rest were GPER-negative in a total of 77 recurrent samples.

### Evaluation of epithelial-mesenchymal transition marker protein expression

Expression of EMT marker proteins (E-cadherin, vimentin, fibronectin) were also detected immunohistochemically in PTs of breast cancer and paired MTs. Expression of membranous E-cadherin, cytoplasmic vimentin and fibronectin was scored using the following system [27]: 0 (no staining); 1 (1 to 25% staining); 2 (26 to 50% staining); 3 (>50% staining).

### Construction of GV115-sh/β1-integrin and siRNA transfection

Four short hairpin RNA (shRNA) sequences targeting the β1-integrin gene and one negative control sequence were designed, and inserted into the lentivirus vector GV115 containing a green fluorescent protein (GFP) reporter. Recombinant lentivirus and control were extracted after transfection of 293 T cells with the recombinant vector and helper vectors. The lentivirus with the best interfering effect as determined by real-time PCR was selected to infect MCF-7R cells. The selected β1-integrin shRNA vector sequence (No. 1756–1) was as follows: forward, 5′-CCGGGAGGAAATGGTGTTTGCAAGTTTCAAGAGAACTTGCAAACACCATTTCCTCTTTTTG-3′; and reverse, 3′-CTCCTTTACCACAAACGTTCAAAGTTCT CTTGAACGTTTGTGGTAAAGGAGAAAAACTTAA-5′. Reagents and technical support were from GeneChem (Shanghai, China). The other β1-integrin shRNA vector sequences and negative control vector sequence are listed in Additional file 1: Table S1. The MCF-7R subclones expressing downregulated β1-integrin were established through infecting the MCF-7R cells with β1-integrin shRNA lentivirus. The same method was used to transduce the negative control virus into cells to control for the impact of the lentivirus vector with the same methods. After infection for 8 hours, the culture medium was refreshed. The cells were transfected with lentivirus vectors for 72 hours and used in subsequent studies. A second shRNA (No. 1755–1) was utilized for data (Additional file 2: Figure S1).

GPER-specific small interfering RNA (siRNA) and the control siRNA sequence (gifts from Dr Gang Tu) were transiently transferred into MCF-7R or SKBR3 cells. The target sequences for GPER siRNA were 5′-GCUGUACAUUGAGCAGAAATT-3′ (A) and 5′-UUUCUG CUCAAUGUACAGCTT-3′ (B). The control siRNA sequence that did not match any known human cDNA was 5′-AAGGTGTCAGAAACTGACGAT-3′. Expression of GPER protein levels was analyzed by Western blotting after transfection.

### Measurement of cell growth

Cells were seeded in 96-well plates at 5 × 10^3^ cells/well. After 24 hours, the cells were treated with different concentrations of TAM for the time indicated, and the medium was renewed on day 3. The vehicle (0.1% ethanol) was used as a control. At the end of the treatment, 20 μl 5 mg/ml MTT was added into the medium and incubated for 4 hours at 37°C. After removing medium, 150 μl MTT solvent (DMSO) was added to each well for 15 minutes and optical density (OD) values were read in a digital spectrophotometer (λ = 490 nm). Each experiment was repeated three times. All the OD values, divided by the average of their controls, were converted to a percentage of the control.

### Wound healing assay and Transwell assays

For wound healing assays, MCF-7R cells were infected with lentivirus vectors targeting β1-integrin or negative control. GFP expression in breast cancer cells at least 2 days after infection was observed by immunofluorescence microscopy. For co-culture preparation, 1 × 10^5^ CAFs were grown to confluence in six-well dishes in complete DMEM for 3 days. Breast cancer cells growing in log-phase were digested with trypsin, and 2 × 10^5^ cells were plated onto the confluent CAFs. At 90% confluence, a pipette tip was used to make a single scratch in the monolayer. Floating cells and debris were removed and cells were incubated with minimum serum medium (DMEM + 0.5% FBS + 100 IU/ml penicillin and 100 mg/ml streptomycin) for 24 hours to allow cell growth and wound closure. Images of the wound were obtained before and after the 24-hour period, then measured using Image J software (National Institutes of Health, Bethesda, MD, USA).

For transwell assays, 2 × 10^4^ breast cancer cells, suspended in 200 μl serum-free medium, were seeded into the upper well of a Boyden chamber with 8-μm pore size filters (Millipore, Darmstadt, Germany). CAFs at approximately 80% confluence were washed and cultured in fresh serum-free DMEM medium for 48 hours. The medium was then collected and filtered for use mixed at a 9:1 ratio with fresh DMEM with 10% FBS as conditioned medium (CM). Normal medium (1% FBS) or CM was added into the lower chamber with or without adding TAM (1 μM) into the upper chamber according to the experimental design. After incubation for 24 hours, cells adhering to the upper surface of the filter were removed using a cotton applicator. After staining with 0.5% crystal violet, the cells that had migrated to the opposite side of the filter were counted. The data represent at least three experiments performed in triplicate (mean ± standard error).

### Reverse transcription and real-time PCR

Total RNA was extracted from MCF-7 and MCF-7R cells using RNAiso reagent (TaKaRa, Dalian, China) according to the manufacturer’s protocol. Total cDNA was synthesized from RNA via a PrimeScript RT reagent Kit (TaKaRa). The expression of β1-integrin was quantified by quantitative real-time PCR performed with the Bio-Rad Miniopticom Real-time PCR system using SYBR® Premix EX Taq™II Kit (TaKaRa). The following primer sequences were used: 5′-CCTACTTCTGCACGATGTGATG-3′ (β1-integrin forward), 5′-CCTTTGCTACGGTTGGTTACATT-3′ (β1-integrin reverse), 5′-TGACTTCAACAGCGACACCCA-3′ (GAPDH forward) and 5′-CACCCTGTTGCTGTAGCCAAA-3′ (GAPDH reverse). All the samples were amplified by real-time PCR twice and expression was normalized to GAPDH.

### Western blotting

Cells were treated as described in the figure legends for various times indicated in the results. Protein cell lysates and Western blotting procedures were performed as described previously [17]. Subcellular protein fractions were extracted using a Cell Membrane Protein Extraction Kit (Beyotime) following the manufacturer’s instructions. Cellular proteins (50 μg) were boiled in SDS-PAGE sample loading buffer and separated by 10% SDS-PAGE, and the specific primary antibody which was targeting each protein was incubated with the membranes at 4°C overnight. Later, the membranes were incubated with appropriate horseradish peroxidase-conjugated secondary antibody and visualized using the chemical luminescence imaging system. OD was analyzed using Image J Software, and results normalized to β-actin were expressed as fold change relative to the control. Each experiment was performed at least three times with representative gels shown.

### Immunofluorescence

Cells were grown on sterile glass coverslips in 24-well plates treated as described in the figure legends. After 24 hours, cells were washed with cold PBS, fixed in 4% paraformaldehyde, permeabilized with 0.2% Triton X-100, and blocked with 5% goat serum. Cells were then incubated with the primary antibody overnight at 4°C, and then with secondary antibody conjugated with FITC or TRITC (1:200; ZsBio). DAPI (Invitrogen) was used to visualize nuclei. The images were viewed using a Nikon Eclipse 80i microscope (Nikon, Tokyo, Japan).

### Statistical analysis

Statistical analysis was performed using SPSS standard version 19.0 software (Chicago, USA). Results are expressed as means ± standard deviation from at least three independent determinations. The Student’s *t*-test was used for single comparisons between two groups, and analysis of variance followed by the Student–Newman–Keuls multiple comparisons test was used for comparison between multiple groups. Values of *P* < 0.05 were considered statistically significant.

## Results

### Consistency in changes in G protein-coupled estrogen receptor, β1-integrin and epithelial-mesenchymal transition marker protein expression in tumors that recurred during treatment with tamoxifen

**Fig. 1:**
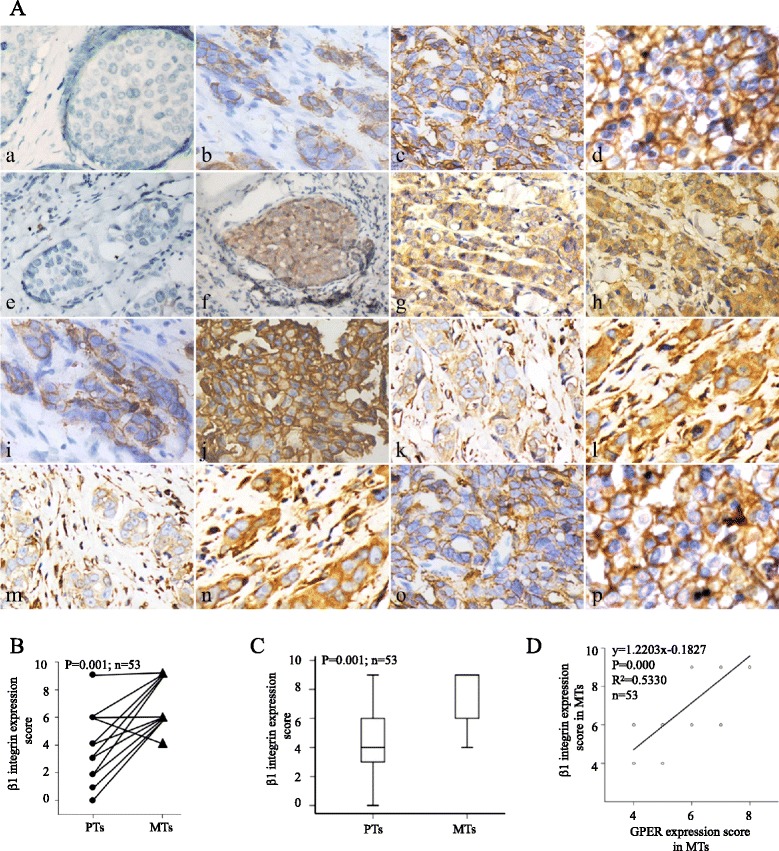
Immunohistochemical staining of β1-integrin, G protein-coupled estrogen receptor (GPER) and epithelial-mesenchymal transition (EMT) marker proteins in breast cancer tissues. The predominant staining pattern of GPER, vimentin and fibronectin was cytoplasmic in carcinoma tissues, whereas β1-integrin and E-cadherin were expressed mainly on the plasma membrane **(A)**. (a-d) β1-integrin immunostaining showing representative negative (a), weak (b), moderate (c) or strong positive plasma membrane staining (d). (e-h) GPER staining showing representative negative (e), weak (f), moderate (g) or strong positive staining (h). (i-n) EMT marker proteins staining showing representative weak staining ((i) E-cadherin (k) vimentin (m) fibronectin) and strong staining ((j) E-cadherin (l) vimentin (n) fibronectin). (o,p) Example of weak β1-integrin staining in primary tumor (PT) (o) and strong β1-integrin expression in the corresponding metastasis (MT) (p) under tamoxifen treatment. Original magnification ×400. Matched change **(B)** and quantitative **(C)** comparison of β1-integrin expression in PTs and their corresponding MTs. The correlation of β1-integrin and GPER expression in MTs revealed through pair-wise scatter plots **(D)**

A total of 77 breast cancer tissues were eligible for analysis according to our previous inclusion criteria [17]; of these, 53 recurrent breast cancer specimens (33 local and 20 distant metastases) were identified as GPER^+^. Among the 53 GPER^+^ specimens, GPER expression was increased in 73.58% (39/53), decreased in 5.66% (3/53) and unchanged in 20.76% (11/53) compared with the matched PTs. All these GPER^+^ MTs and the paired PTs were also used to determine β1-integrin expression. β1-integrin was shown to be expressed predominantly on the plasma membrane and GPER in the cell cytoplasm (Fig. 1a).

Our previous report showed that the mean immunohistochemical score for GPER in MTs (6.23 ± 0.91) was significantly increased compared with that in PTs (3.46 ± 1.07; *P* = 0.001). To investigate the potential relationship among GPER, β1-integrin and tamoxifen resistance, β1-integrin expression was scored in PTs and the paired MTs. β1-integrin expression was increased in 79.2% (42/53), decreased in 5.66% (3/53) and unchanged in 15.1% (8/53) of these 53 GPER^+^ tumors that relapsed during tamoxifen treatment (Fig. 1b). The mean immunohistochemical score for β1-integrin was 4.60 ± 1.96 in PTs and 7.42 ± 1.69 in MTs (Fig. 1c, *P* = 0.001). Among the 53 MTs (GPER^+^), β1-integrin expression was positively correlated with GPER expression (Fig. 1d, *R*^2^ = 0.5330, *P* = 0.000).

To determine if the epithelial characteristics of breast cancer cells exhibited transitions during clinical treatment with tamoxifen, the expression of EMT marker proteins (E-cadherin, vimentin and fibronectin) was investigated in these 53 GPER^+^ MTs and paired PTs. Representative weak staining and strong staining are shown in Fig. 1a. Significant increases in cytoplasmic vimentin (*P* = 0.001) and fibronectin (*P* = 0.011) expression scores were observed (mean immunohistochemical scores: vimentin, 0.66 ± 0.586 in PTs and 1.38 ± 0.790 in MTs; fibronectin, 0.60 ± 0.631 in PTs and 1.30 ± 0.822 in MTs). However, the mean immunohistochemical score for E-cadherin in this group of patients was 2.40 ± 0.716 in PTs and 2.25 ± 0.875 in the recurrent lesions (*P* = 0.059).

### G protein-coupled estrogen receptor may mediate increased β1-integrin expression in tamoxifen-resistant breast cancer cells

**Fig. 2:**
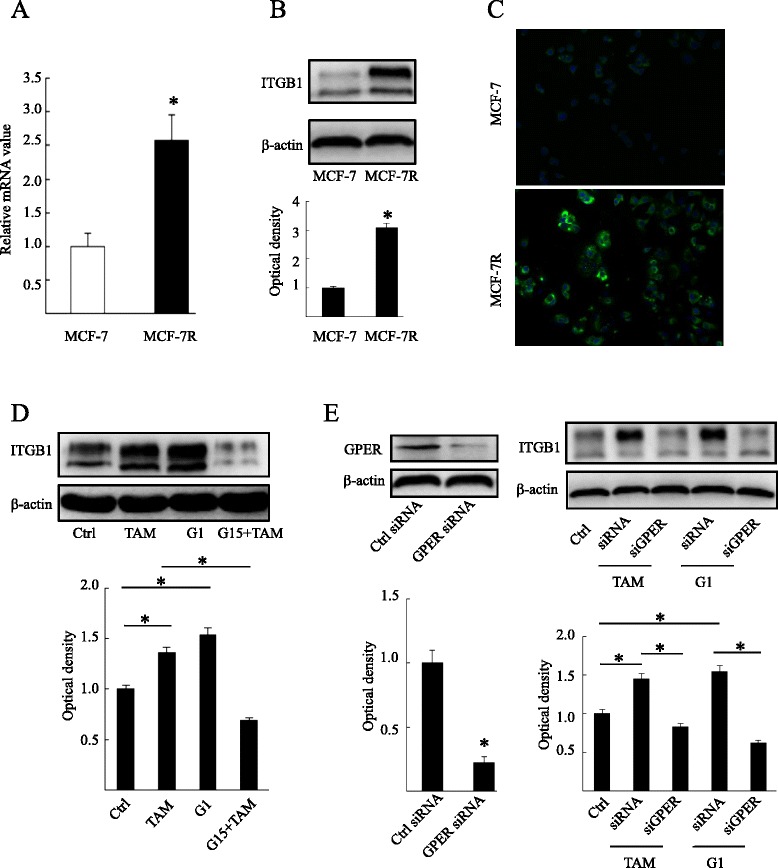
Tamoxifen-induced β1-integrin expression is mediated by G protein-coupled estrogen receptor in MCF-7R cells. MCF-7 cells and MCF-7R cells were analyzed for the presence of β1-integrin by (**a**) RT-PCR, (**b**) Western blotting and (**c**) immunofluorescence. The relative fold-change was compared with MCF-7 cells (*P < 0.05, Student’s *t*-test). (**d**) β1-integrin was detected through Western blotting. MCF-7R cells were treated with ethanol (control), G1 (10 nM) and TAM (1 μM) alone or in combination with the G protein-coupled estrogen receptor (GPER) antagonist G15 (1 μM) for 24 hours. (**e**) Western blotting analysis was used to test the effect of GPER-specific siRNA on GPER (left panel) and β1-integrin (right panel) levels in MCF-7R cells. Each experiment was repeated at least three times. Results are shown as fold-changes in optical density compared with the control, and normalized to β-actin. *P < 0.05. Ctrl, control; ITGB1, β1-integrin; TAM, 4-hydroxytamoxifen

It has been reported that GPER plays an important role in tamoxifen resistance in breast cancer [15, 17]. To explore the underlying mechanisms, we previously screened the target genes of GPER via microarray analysis in breast cancer cells, and sixty target genes were identified according to a rigorous screening criteria (data unpublished; Additional file 3: Table S2). One of these genes, β1-integrin, has been implicated in therapeutic resistance in multiple solid cancer models [20, 21]. To determine the role of β1-integrin in tamoxifen resistance of breast cancer cells, we first evaluated its expression in tamoxifen-sensitive MCF-7 cells (MCF-7) and tamoxifen-resistant MCF-7 cells (MCF-7R). The increased β1-integrin expression in MCF-7R cells compared to that in MCF-7 cells was confirmed by quantitative RT-PCR, immunofluorescence and Western blotting analyses (Fig. 2). Moreover, the β1-integrin protein was upregulated by the GPER agonist G1 and TAM, while the GPER-specific antagonist G15 and GPER-specific siRNA abolished the agonistic action of TAM in MCF-7R cells (Fig. 2d,e), indicating that β1-integrin expression induced by TAM is mediated by GPER. Interestingly, we saw a similar finding in another GPER-positive cell line SKBR3 (Additional file 2: Figure S2). It should be noted that G15 and GPER-specific siRNA alone had no significant impact on β1-integrin expression (data not shown).

### The EGFR/ERK signaling pathway is directly responsible for increased β1-integrin expression in MCF-7R cells

**Fig. 3:**
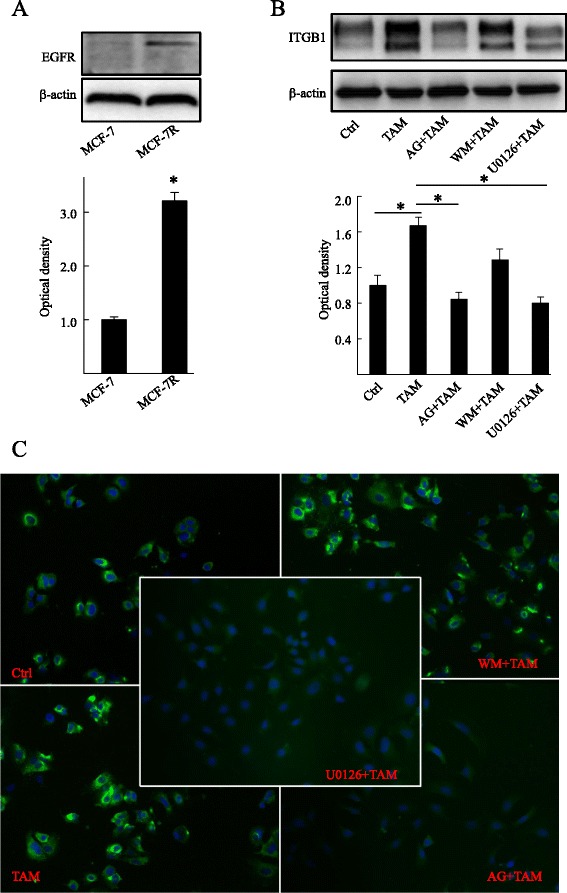
Tamoxifen induces β1-integrin expression through a mechanism that involves the EGFR and MAPK/ERK signaling pathways. (**a**) Levels of EGFR were detected by Western blotting using specific antibodies in cells. MCF-7R cells were treated with ethanol (control), TAM (1 μM) alone or with the addition of the specific signaling pathway inhibitor targeting to EGFR (AG1478; 6 μM; AG), MAPK/ERK (U0126; 10 μM) or PI3K (Wortmannin; 10 μM; WM). β1-integrin expression was detected by (**b**) Western blotting and (**c**) immunofluorescence analyses. Each experiment was repeated at least three times. Western blotting results are shown as fold-changes in optical density compared with the control, and normalized to β-actin. *P < 0.05. Ctrl, control; ITGB1, β1-integrin; TAM, 4-hydroxytamoxifen

EGFR is involved in the induction of tamoxifen resistance and is known to activate the MAPK/ERK and PI3K/AKT pathways in breast cancer cells [28, 29]. It was found that EGFR was highly expressed in MCF-7R cells compared with MCF-7 cells (Fig. 3a). Moreover, it has been previously demonstrated that TAM activates the MAPK/ERK and PI3K/AKT pathways through the GPER/EGFR signaling pathway in MCF-7R cells [15, 17]. To investigate whether these pathways are implicated in inducing β1-integrin expression, MCF-7R cells were treated with TAM alone or with the addition of the signaling pathway inhibitors. The specific EGFR inhibitor AG1478 (6 μM; Sigma-Aldrich) and the MAPK/ERK inhibitor U0126 (10 μM; Beyotime) abolished the agonistic effect of TAM (1 μM) on the expression of β1-integrin, while the PI3K inhibitor Wortmannin (1 μM; Sigma-Aldrich) did not (Fig. 3b,c). None of the inhibitors had any effect on β1-integrin expression when used alone (data not shown). Our results suggest that TAM stimulates β1-integrin expression in MCF-7R cells via the GPER/EGFR/ERK signaling pathway.

### Blockage of β1-integrin partially rescues the sensitivity of MCF-7R cells to tamoxifen

**Fig. 4:**
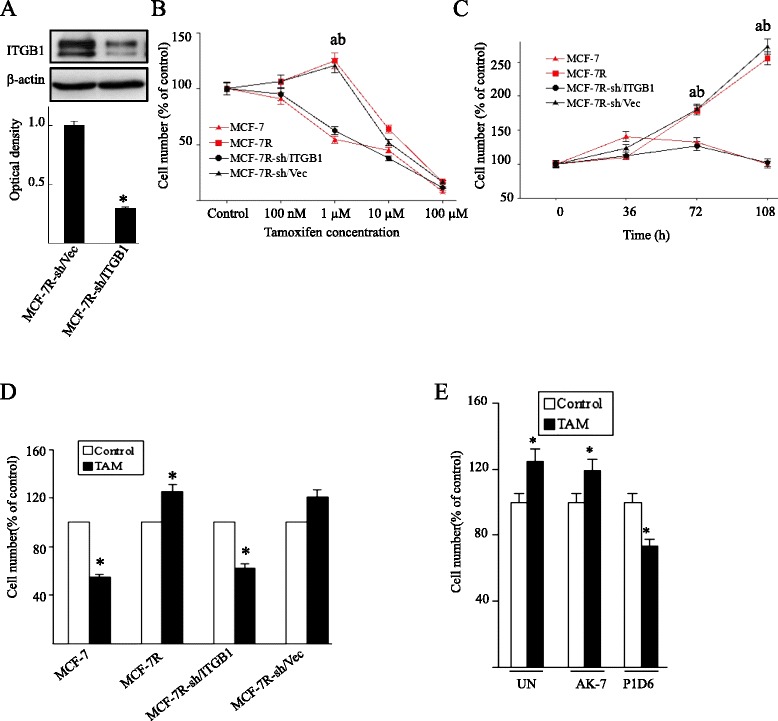
β1-integrin silencing restores the tamoxifen sensitivity in MCF-7R cells. (**a**) The effect of a lentivirus-mediated short hairpin RNA (shRNA) vector targeting β1-integrin was measured by Western blotting in MCF-7R cells. Results are shown as fold-changes in optical density compared with the control (sh/Vec), and normalized to β-actin. *P < 0.05, versus control. Cells were treated with (**b**) the indicated concentrations of TAM for 108 hours or (**c**) with therapeutic concentrations of TAM (1 μM) for the indicated time. The data represent means ± standard deviation from three different experiments (^a^ *P* < 0.05, MCF-7R cells versus MCF-7 cells; ^b^ *P* < 0.05, MCF-7R-sh/Vec versus MCF-7R-sh/ITGB1). (**d**) Cells were treated with 1 μM TAM and counted after 108 hours. (**e**) Cell numbers were measured in MCF-7R cells that were left untreated (UN) or pretreated with the specific inhibitory antibody for α2β1-integrin (AK-7; 20 μg/ml) or α5β1-integrin (P1D6; 10 μg/ml) before TAM stimulation. Results are expressed as means ± standard deviation of three independent experiments. *P < 0.05, versus each control. ITGB1, β1-integrin; MCF-7R-sh/ITGB1, MCF-7R cells infected with lentivirus vector targeting β1-integrin; MCF-7R-sh/Vec, MCF-7R cells infected with negative control lentivirus (control); TAM, 4-hydroxytamoxifen

The effects of β1-integrin silencing in MCF-7R cells were investigated to determine the role of β1-integrin in tolerance to TAM. The expression of β1-integrin protein was silenced with a lentivirus-mediated shRNA vector (Fig. 4a). MCF-7R subclones with silenced β1-integrin (MCF-7R-sh/ITGB1) were also established. Cell growth following treatment with TAM (0 to 100 μM) for 108 hours was determined using MTT assays. As expected, low TAM concentrations (0 to 1 μM) inhibited MCF-7 cell growth and stimulated MCF-7R cell growth. When the concentration of TAM was elevated to 100 μM, there was no obvious difference in cell survival, suggesting off-target effects of TAM at high concentrations. Excitingly, inhibition of β1-integrin expression in MCF-7R restored the TAM inhibitory effect (Fig. 4b). Additionally, investigation of the effect of therapeutic concentrations of TAM (1 μM) on cell growth over time showed that the inhibitory effects of TAM were first apparent at 72 hours and were more significant at 108 hours (Fig. 4c,d). Taken together, these observations demonstrate that β1-integrin plays an important role in acquired tamoxifen resistance.

In some instances, GPER enhances α5β1-integrin-mediated fibronectin matrix assembly [30] that promotes cellular adhesion, haptotaxis, and survival. Therefore, we examined whether α5β1-integrin was involved in tamoxifen sensitivity. Pretreatment of MCF-7R cells with the function-blocking antibody specific for α5β1-integrin (P1D6), but not α2β1-integrin (AK-7), partially rescued the sensitivity of MCF-7R cells to tamoxifen (Fig. 4e).

### The effect of cancer-associated fibroblasts on cancer cell migration is abolished by inhibition of β1-integrin

**Fig. 5:**
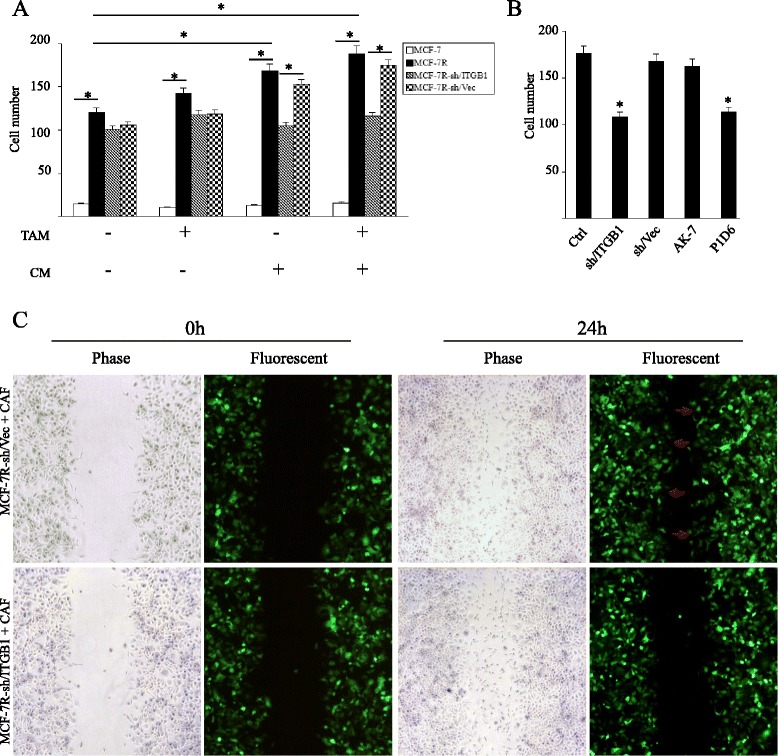
β1-integrin mediates the effect of cancer-associated fibroblasts on breast cancer cell migration. Cell migratory capacity was assessed in Transwell assays and wound healing assay. (**a**) *In vitro* Transwell assays were performed using cancer-associated fibroblast (CAF)-conditioned medium (CM) or normal medium with or without the addition of TAM (1 μM). (**b**) The number of cells with indicated pretreatment that migrated toward the lower wells of the transwell containing CM was counted. Each experiment was repeated at least three times. *P < 0.05. (**c**) The capacity of cells to migrate to fill a scratched area devoid of cells was assessed in co-cultures of breast cancer cells and CAFs. Before co-culture, breast cancer cells were infected with lentivirus carrying a green fluorescent protein reporter gene to distinguish CAFs under a fluorescence microscope. Fluorescence photomicrographs revealed that MCF-7R-sh/Vec showed enhanced cell migration ability into the scratched area (arrows) when compared with MCF-7R-sh/ITGB1. Ctrl, control; ITGB1, β1-integrin; MCF-7R-sh/ITGB1, MCF-7R cells infected with lentivirus vector targeting β1-integrin; MCF-7R-sh/Vec, MCF-7R cells infected with negative control lentivirus (control); TAM, 4-hydroxytamoxifen

It has been reported that breast cancer cells display enhanced mobility following the acquisition of tamoxifen resistance [12, 13]. The effects of β1-integrin inhibition on cell migration were investigated using *in vitro* Transwell assays. Unexpectedly, inhibition of β1-integrin expression in MCF-7R cells had no significant effect on cell migration regardless of the presence or absence of TAM (Fig. 5a). Integrins function as a critical link between tumor cells and the surrounding microenvironment [31]; therefore, we investigated whether CAFs promoted breast cancer cell migration and investigated the effects of β1-integrin inhibition on this process. Interestingly, CAF-CM significantly increased the Transwell migration of MCF-7R cells compared with MCF-7 cells. Furthermore, the function of CM was notably reversed by β1-integrin silence and the α5β1-integrin-inhibitory antibody P1D6 (Fig. 5a,b). Additionally, CM enhanced the cell migration of MDA-MB-231 cells, but not MDA-MB-468 cells, through β1-integrin signaling (Additional file 2: Figure S3).

To further confirm the effects of CAF-stimulation on the tumor cell migration, a wound healing assay was performed under direct co-culture conditions. The size of the scratched area narrowed and almost closed within 24 hours when breast cancer cells were co-cultured with CAFs, observed by phase-contrast microscopy. However, fluorescent photomicrographs showed that the breast cancer cells expressing GFP migrated into the scratched area. Our study found that, although CAFs nearly closed the wound area in the two groups, the MCF-7R cells with silenced β1-integrin (MCF-7R-sh/ITGB1) showed relatively weak migratory capacity compared with the control group (MCF-7R-sh/Vec) (Fig. 5c).

### The β1-integrin downstream kinases FAK and Src are activated on acquisition of tamoxifen resistance

**Fig. 6:**
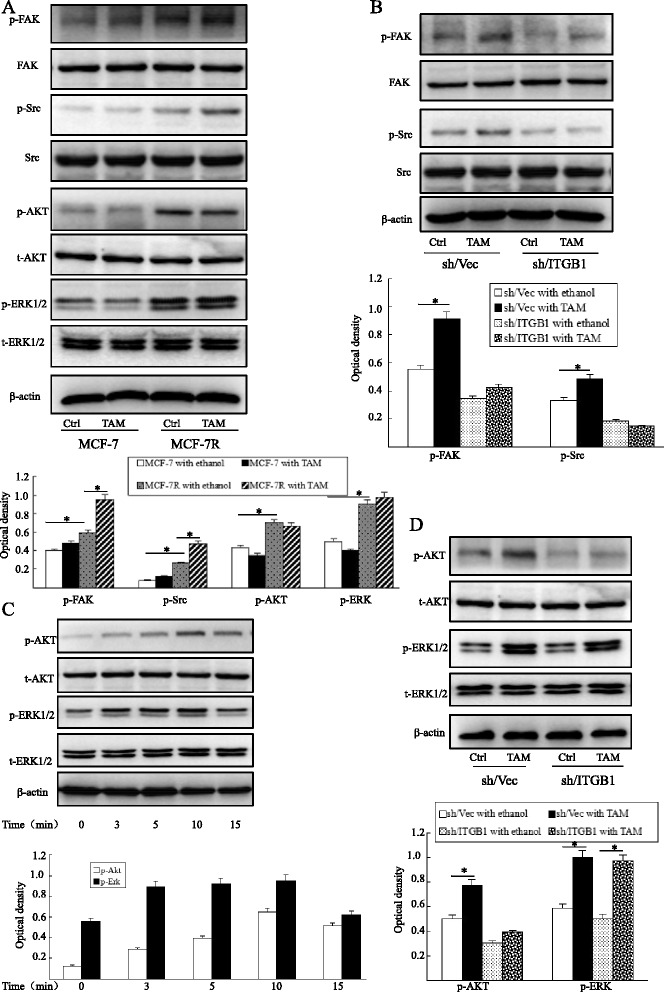
Tamoxifen induces the activation of FAK, Src and AKT through β1-integrin in MCF-7R cells. (**a**) Cells were treated with or without TAM (1 μM) for 24 hours prior to the analysis of protein expression by Western blotting. (**b**) The effects of TAM (1 μM for 24 hours) on pFAK and pSrc were investigated by Western blotting in MCF-7R with silenced β1-integrin (sh/ITGB1) and control cells (sh/Vec). (**c**) MCF-7R cells were cultured with TAM (1 μM) for the indicated time, then AKT and ERK1/2 activity was measured by Western blotting using specific antibodies. (**d**) MCF-7R cells with silenced β1-integrin (sh/ITGB1) and control cells (sh/Vec) were treated with TAM (1 μM) for 10 minutes and then analyzed by Western blotting. Each experiment was repeated at least three times. Results are shown as fold-changes in optical density compared with total levels, and normalized to β-actin. *P < 0.05. Ctrl, control; ITGB1, β1-integrin; sh/ITGB1, MCF-7R cells infected with a lentivirus vector targeting β1-integrin; sh/Vec, MCF-7R cells infected with negative control lentivirus; TAM, 4-hydroxytamoxifen

The MCF-7R cell line was developed through long-term exposure to TAM. Immunoblot analysis of the MCF-7R revealed obvious elevation of phosphorylated (p)AKT and ERK in MCF-7R compared with untreated parental MCF-7 cells (Fig. 6a). In MCF-7R cells, the levels of the β1-integrin downstream kinases pFAK (Y397) and pSrc (Y418) were also increased significantly (Fig. 6a). Furthermore, cells were treated with 1 μM TAM for 24 hours, and FAK and Src activity was then determined by Western blotting. TAM promoted phosphorylation of FAK at Y397 and Src at Y418 in MCF-7R cells but not in MCF-7 cells, while total FAK and Src were unaffected (Fig. 6a), which was consistent with CM treatment (data not shown).

The levels of pFAK and pSrc were decreased modestly and no changes in total levels were detected in β1-integrin-downregulated MCF-7R cells. Additionally, the positive effect of TAM on FAK and Src activity was suppressed by β1-integrin inhibition (Fig. 6b).

### Activation of the AKT signaling pathway in response to TAM is mediated by β1-integrin in tamoxifen-resistant MCF-7 cells

Activation of AKT and MAPK is known to play a pivotal role in tamoxifen resistance. Also, ERK1/2, part of a major MAPK pathway cascade, mediates mitogenesis in hormone-sensitive breast cancer cells. We tested the effect of TAM on phosphorylation of AKT and ERK in MCF-7R cells and compared this to control cells. Although there was no marked alteration in AKT and EKR1/2 activity at 24 hours after TAM treatment (Fig. 6a), TAM mediated their rapid phosphorylation with peak increases at 10 minutes in MCF-7R cells (Fig. 6c). However, the activation of AKT and EKR1/2 was much later and weaker in MCF-7 cells (data not shown). Moreover, our data showed that activation of the AKT signaling pathway in response to TAM is mediated by β1-integrin in MCF-7R cells (Fig. 6d). Interestingly, Huang and colleagues found similar results in lapatinib-resistant HER2^+^/ER^+^ and Her2^+^/ER^−^ cell lines [21].

### β1-integrin is involved in the cancer-associated fibroblast-induced epithelial-mesenchymal transition process in MCF-7R cells

**Fig. 7:**
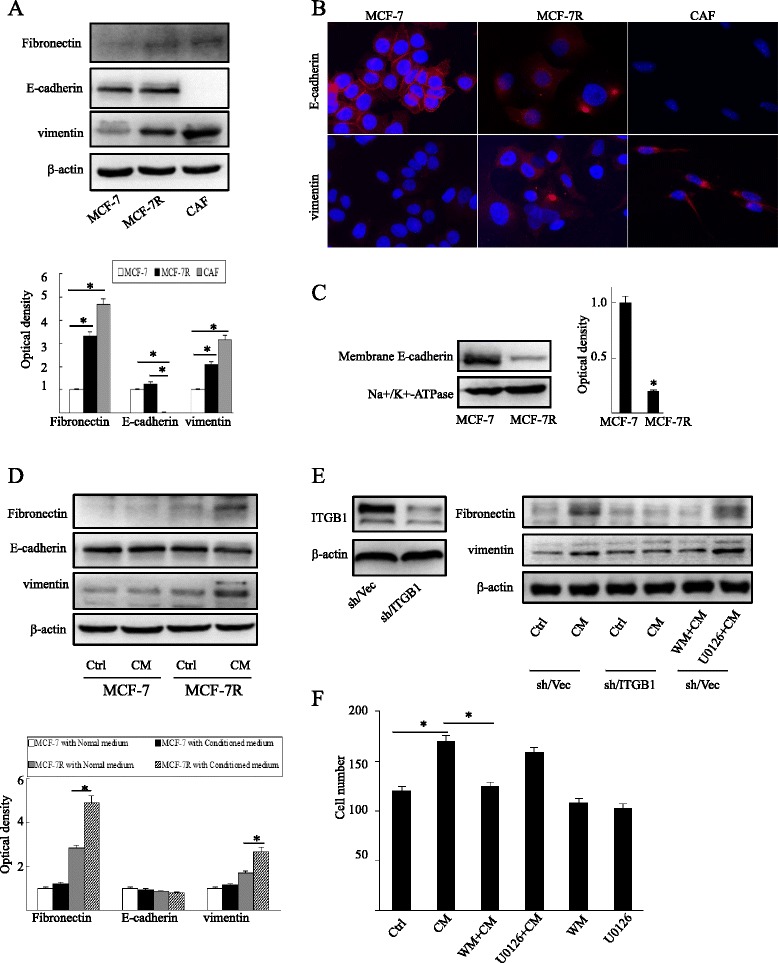
β1-integrin is implicated in the epithelial-mesenchymal transition process induced by cancer-associated fibroblasts in MCF-7R cells. (**a**) Equal amounts of total cell protein were separated by SDS-PAGE and subsequently probed with antibodies against E-cadherin, fibronectin and vimentin. (**b**) Localization and expression of E-cadherin and vimentin (red) in MCF-7, MCF-7R, and cancer-associated fibroblast (CAF) cell lines were revealed by immunofluorescence staining. The nucleus was stained blue with DAPI. Original magnification: ×200. (**c**) Levels of membrane E-cadherin were detected by Western blotting using specific antibodies. Na^+^/K^+^-ATPase was used for normalization. (**d**) Cells were cultured in CAF-conditioned medium (CM) or normal medium for 48 hours, and the expression of E-cadherin, fibronectin and vimentin was then determined by Western blotting. (**e**) β1-integrin expression in MCF-7R cells was blocked by lentivirus vector transfection (left panel). Cells were left untreated (control; Ctrl) or treated with CM for 48 hours, or pretreated with the PI3K inhibitor Wortmannin (10 μM; WM) or the MAPK/ERK inhibitor U0126 (10 μM) prior to CM treatment, and then cell lysates were probed with antibodies against fibronectin and vimentin (right panel). (**f**) Cell migration of MCF-7R cells with indicated pretreatments was measured through Transwell assays. Each experiment was repeated at least three times. Western blotting results are shown as fold-changes in optical density compared with the control, and normalized to β-actin. *P < 0.05. ITGB1, β1-integrin; sh/ITGB1, MCF-7R cells infected with a lentivirus vector targeting β1-integrin; sh/Vec, MCF-7R cells infected with negative control lentivirus

Acquired chemotherapeutic resistance is accompanied by an aggressive, invasive phenotype as a result of EMT [8, 9]. In the current study, Western blotting analysis showed increased expression of vimentin and fibronectin in MCF-7R cells compared with that in MCF-7 cells. However, there was no significant difference in the level of E-cadherin expression (Fig. 7a). These results were confirmed by immunofluorescence staining. Interestingly, although E-cadherin expression was not decreased in association with tamoxifen resistance, it was found to be translocated from the cell membrane to the cytoplasm (Fig. 7b,c). Our data suggest that MCF-7R cells display EMT-like properties compared to MCF-7 cells *in vitro*.

The microenvironment of cancer cells, which contains several distinct stromal cell types, participates in both tumor progression and the response to treatment [32, 33]. CAFs, which are one of the most crucial components of the tumor microenvironment, possess biological properties and functions that are distinct from those of normal fibroblasts [34]. It has been reported that CAFs promote cancer cell growth and migration through an involvement in angiogenesis and EMT [35, 36]. To determine whether CAFs have the capacity to regulate tumor progression, MCF-7 cells and MCF-7R cells were cultured in the CM. The expression of fibronectin and vimentin was significantly elevated in MCF-7R cells cultured in CM compared to those cultured in normal medium. However, CM exerted a marginal effect on MCF-7 cells (Fig. 7d). Moreover, the assumptive E-cadherin decrease was not observed in breast cancer cells cultured in CM. To determine if β1-integrin modulates the CAF-induced EMT process, we tested the effect of CM on MCF-7R cells with silenced β1-integrin (MCF-7R-sh/ITGB1). The influence of CM on the induction of EMT in MCF-7R was found to be neutralized by β1-integrin inhibition (Fig. 7e). Furthermore, PI3K inhibitor Wortmannin reversed the stimulatory effect of CM (Fig. 7e,f). To identify the underlying EMT-inducing factor in CM, we investigated whether the extracellular matrix (ECM) component fibronectin, the main ligand of α5β1-integrin, was involved in the process. Interestingly, fibronectin had a similar impact on EMT induction in MCF-7R cells (Additional file 2: Figure S4).

## Discussion

The ability to overcome endocrine resistance is one of the important aims for hormone-dependent breast cancer patients [37]. GPER, a novel membrane-bound estrogen receptor, has been demonstrated to contribute to the development of tamoxifen resistance [15, 17]. However, the specific mechanism is less clearly elucidated. In the present study, we found that tamoxifen resistance initiated by GPER is associated with CAF-derived tumor microenvironment/β1-integrin interaction and accompanied by EMT. More interestingly, tamoxifen-resistant MCF-7 cells (MCF-7R) were shown to be more susceptible to EMT than MCF-7 cells in the presence of stromal CAFs, which are one of the predominant members in the tumor microenvironment, in a β1-integrin-dependent manner.

GPER, whose action differs from the classical nuclear estrogen receptors (ERα, ERβ), acts as an independent ER in breast cancer cells and has been implicated in mediating both rapid and transcriptional events in response to estrogen under certain circumstances [16, 24, 38]. Although no increased basal expression of GPER in MCF-7R cells is observed when compared to MCF-7 cells, translocation of GPER from the cytoplasm to cell membrane reinforces the crosstalk between GPER and EGFR during long-term treatment with tamoxifen in breast cancer cells [15, 17]. This crosstalk is followed by phosphorylation of MAPK and AKT, which subsequently stimulates gene transcription and development of tamoxifen resistance [15].

β1-integrin is an important member of a family of heterodimeric transmembrane adhesion proteins [6]. Several recent studies have convincingly demonstrated that β1-integrin is involved in therapeutic resistance in various tumor types [20, 21, 39, 40]. Indeed, β1-integrin was identified as one of the GPER target genes in MCF-7R cells in our previous work (unpublished data). Here, we found that enhanced β1-integrin expression in MCF-7R cells was mediated via GPER/EGFR/ERK signaling; the significantly increased β1-integrin in metastases compared with the matched PTs was closely GPER-related. This was supported by a proteomic analysis of acquired tamoxifen resistance in MCF-7 cells [41]. Additionally, as downstream kinases of β1-integrin, Src and FAK kinase activity was significantly enhanced and involved in cellular invasion and motility in tamoxifen-resistant breast cancer cells [42, 43]. These findings suggest that GPER-mediated tamoxifen resistance is associated with the enhanced β1-integrin and its downstream signaling activation in breast cancer cells.

In this study, MCF-7R cells displayed EMT-like properties compared to MCF-7 cells *in vitro* and clinical tamoxifen-resistant breast tumor tissues. Although no significant total E-cadherin expression was detected, decreased membrane E-cadherin was detected in MCF-7R cells. It has been shown that E-cadherin internalization is a key step in its dysfunction [12, 44], indicating EMT is responsible for the adverse phenotype in MCF-7R cells. β1-integrin is known to be implicated in the malignant tumor characteristics, such as increased migration and invasion, in a variety of tumor types [18, 20, 45, 46]. Interestingly, we observed that a potential ability for the development of EMT was acquired after MCF-7R cells were exposed to CAFs, CAF-derived CM, or fibronectin treatment. Indeed, some of elements in ECM, such as collagen I, collagen III and collagen IV were reported to protect tumor cells from chemotherapy attraction [32, 47, 48]. ECM acts as a substrate to which cells adhere and serves as a reservoir for growth factors [49]. The ECM component fibronectin is the crucial ligand of α5β1-integrin [30, 31]; the cross-talk between fibronectin and β1-integrin could activate a downstream signaling pathway network in EMT transition (our unpublished data). Several previous reports demonstrated that EMT characteristics might confer a chemo-drug resistant potential in tumor cells [10, 14]. In the current study, we further disclosed that CAF-derived fibronenctin conferred EMT phenotype to MCF-7R cells through the α5β1-integrin/PI3K/AKT signaling axis. These data have highlighted the significance of the tumor microenvironment in tamoxifen resistance and tumor progression. Our findings support the notion that therapy regimens abrogating the complex interactions of the carcinomas with the tumor microenvironment could be a future alternative to overcome tamoxifen resistance in breast cancer, and β1-integrin may be a promising target.

## Conclusions

**Fig. 8:**
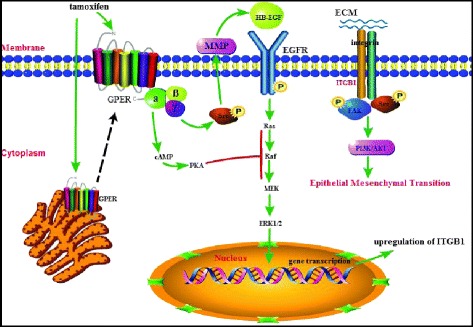
Illustration depicting the role of G protein-coupled estrogen receptor and β1-integrin in the epithelial-mesenchymal transition process induced by cancer-associated fibroblasts. Long-term exposure to tamoxifen promotes the translocation of G protein-coupled estrogen receptor (GPER) to the cell membrane, which enhances the crosstalk between GPER and epidermal growth factor receptor (EGFR). The Ga subunit of GPER is responsible for the increase in cAMP generation in breast cancer cells and cAMP attenuates ERK1/2 activity by suppressing protein kinase A (PKA) on Raf, whereas cAMP production triggered by GPER is disorganized upon acquisition of tamoxifen resistance leading to the increased activation of ERK1/2. Tamoxifen, as an agonist for GPER, stimulates the induction of β1-integrin expression through the GPER/EGFR/ERK signaling pathway. Upregulation of β1-integrin is accompanied by the activation of the β1-integrin signaling pathway, which is measured by FAK and Src activity. Conditioned medium (CM) produced by cancer-associated fibroblasts (CAFs) induces epithelial-mesenchymal transition (EMT) through the activation of PI3K/AKT, with the involvement of β1-integrin. Targeted therapy with β1-integrin could reverse the stimulatory effect of CAFs in the tumor microenvironment on cell motility. ECM, extracellular matrix; ITGB1, β1-integrin; MMP, matrix metalloproteinase

In summary, our data provide a novel insight into understanding the role of the tumor microenvironment in tamoxifen-resistant breast cancer. Long-term tamoxifen treatment facilitates translocation of GPER to cell membranes, resulting in aberrant activation of the EGFR/ERK signaling pathway and upregulation of β1-integrin expression which is responsible for the enhanced communications between tumor cells and the tumor microenvironment (Fig. 8). Blockage of GPER/EGFR/ERK/β1-integrin signaling may be a potential target in enhancing their sensitivity for tamoxifen-resistant breast cancer patients. However, it is undoubted that further investigations, including *in vivo* experiments and prospective clinical studies, are needed.

## Additional files

Additional file 1: Table S1. Sequences of shRNA vectors targeting β1-integrin gene and one negative control. Additional file 2: Figure S1. A second shRNA sequence applied to MCF-7R cells confirms the important role of β1-integrin in tamoxifen resistance of breast cancer. The effect of this shRNA on β1-integrin expression (A), cell growth (B) and cell migration (C) was detected with the same method as previously described. **P* < 0.05. **Figure S2.** GPER mediates the upregulation of β1-integrin induced by tamoxifen in SKBR3 cells. (A) The expression of GPER was knockdown by GPER-specific siRNA transfection in SKBR3 cells. **P* < 0.05, versus control. (B) Western blotting analysis was used to test the effect of TAM on the level of β1-integrin treated with or without GPER specific siRNA transfection in SKBR3 cells. **P* < 0.05. **Figure S3.** β1-integrin governs the effect of CAFs on MDA-MB-231 cells migration. (A) β1-integrin expression was determined in cells. (B) Expression of β1-integrin was silenced by lentivirus-mediated shRNA in MDA-MB-231 cells. **P* < 0.05, versus control. (C) *In vitro* Transwell assays were performed using CAF-conditioned medium (CM) or normal medium in MDA-MB-468, MDA-MB-231, MDA-MB-231 cells with silenced β1-integrin (MDA-MB-231-sh/ITGB1) and control cells (MDA-MB-231-sh/Vec). **P* < 0.05. **Figure S4.** Fibronectin induces the EMT process through α5β1-integrin and PI3K/AKT signaling in MCF-7R cells. MCF-7R cells were treated with fibronectin (30 μg/ml; FN) for 48 hours, or pretreated with or without α5β1-integrin inhibitory antibody P1D6 (10 μg/ml), the PI3K inhibitor Wortmannin (10 μM; WM), and the MAPK/ERK inhibitor U0126 (10 μM) prior to FN treatment, and then cell lysates were probed with antibodies against fibronectin and vimentin. **P* < 0.05. Additional file 3: Table S2. List of 60 target genes of GPER. The target genes were picked out through cDNA microarray analysis (unpublished data) according to the rigorous screening criteria: at least two-fold increase in MCF-7R cells compared with MCF-7 cells, at least 1.25-fold induction by TAM, at least 1.5-fold induction by G1, and at least 50% suppression of TAM effect by G15 in MCF-7R cells.

## Abbreviations

CAF: cancer-associated fibroblast
CM: conditioned medium
DMEM: Dulbecco's modified Eagle's medium
ECM: extracellular matrix
EGFR: epidermal growth factor receptor
EMT: epithelial-mesenchymal transition
ER: estrogen receptor
FBS: fetal bovine serum
GFP: green fluorescent protein
GPER: G protein-coupled estrogen receptor
MT: metastasis
OD: optical density
PT: primary tumor
shRNA: short hairpin RNA
siRNA: small interfering RNA
