# Supplementary material for: Acquisition of epithelial-mesenchymal transition phenotype in the tamoxifen-resistant breast cancer cell: a new role for G protein-coupled estrogen receptor in mediating tamoxifen resistance through cancer-associated fibroblast-derived fibronectin and β1-integrin signaling pathway in tumor cells
Source: Breast Cancer Res. 2015 May 21;17(1):69. doi: 10.1186/s13058-015-0579-y (PMC4453053; doi:10.1186/s13058-015-0579-y)
Supplement: Additional file 1: Table S1. — Sequences of shRNA vectors targeting β1-integrin gene and one negative control. [file 13058_2015_579_MOESM1_ESM.pdf]

Table S1

NO.	5’	STEM	Loop	STEM	3’
ITGB1-RNAi (1753-1)-a	Ccgg	aaGACGATTTGGAGAATGTAA	TTCAAGAGA	TTACATTCTCCAAATCGTCtt	TTTTTg
ITGB1-RNAi (1753-1)-b	aattcaaaaa	aaGACGATTTGGAGAATGTAA	TCTCTTGAA	TTACATTCTCCAAATCGTCtt	
ITGB1-RNAi (1754-2)-a	Ccgg	gaGGCTCCAAAGATATAAAGA	TTCAAGAGA	TCTTTATATCTTTGGAGCCtc	TTTTTg
ITGB1-RNA (1754-2)-b	aattcaaaaa	gaGGCTCCAAAGATATAAAGA	TCTCTTGAA	TCTTTATATCTTTGGAGCCtc	
ITGB1-RNAi (1755-1)-a	Ccgg	aaGTCAGCAGTAGGAACATTA	TTCAAGAGA	TAATGTTCCTACTGCTGACtt	TTTTTg
ITGB1-RNAi (1755-1)-b	aattcaaaaa	aaGTCAGCAGTAGGAACATTA	TCTCTTGAA	TAATGTTCCTACTGCTGACtt	
ITGB1-RNAi (1756-1)-a	Ccgg	gaGGAAATGGTGTTTGCAAGT	TTCAAGAGA	ACTTGCAAACACCATTTCCtc	TTTTTg
ITGB1-RNAi (1756-1)-b	aattcaaaaa	gaGGAAATGGTGTTTGCAAGT	TCTCTTGAA	ACTTGCAAACACCATTTCCtc	
NC-a	CCGG	TTCTCCGAACGTGTCACGT	TTCAAGAGA	ACGTGACACGTTCGGAGAA	TTTTTg
NC-b	AATTCAAAAA	TTCTCCGAACGTGTCACGT	AAGTTCTCT	ACGTGACACGTTCGGAGAA	
