# Supplementary figures and images for: Acquisition of epithelial-mesenchymal transition phenotype in the tamoxifen-resistant breast cancer cell: a new role for G protein-coupled estrogen receptor in mediating tamoxifen resistance through cancer-associated fibroblast-derived fibronectin and β1-integrin signaling pathway in tumor cells
Source: Breast Cancer Res. 2015 May 21;17(1):69. doi: 10.1186/s13058-015-0579-y (PMC4453053; doi:10.1186/s13058-015-0579-y)

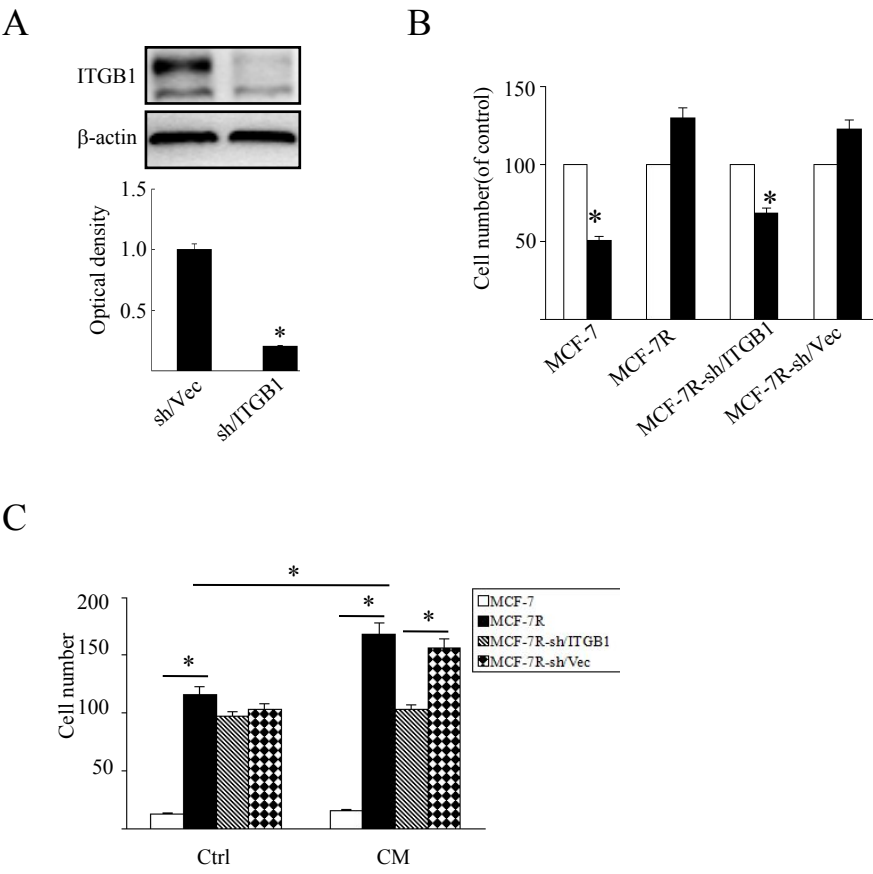

Figure S1

A

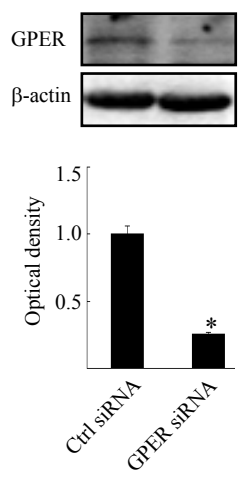

B

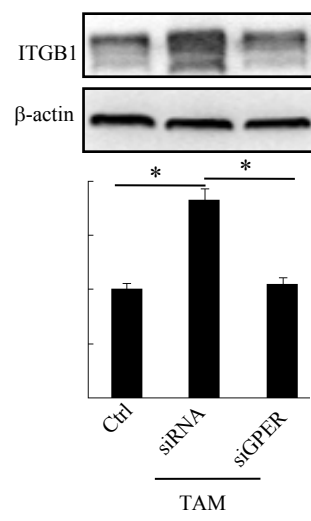

Figure S2

A

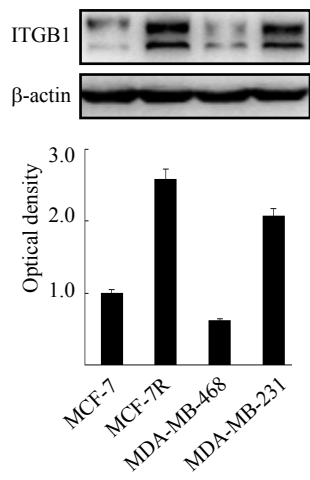

B

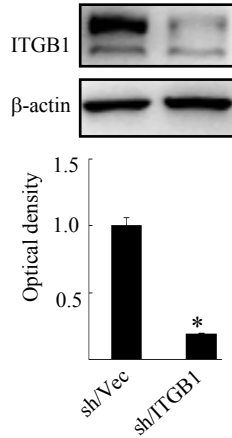

C

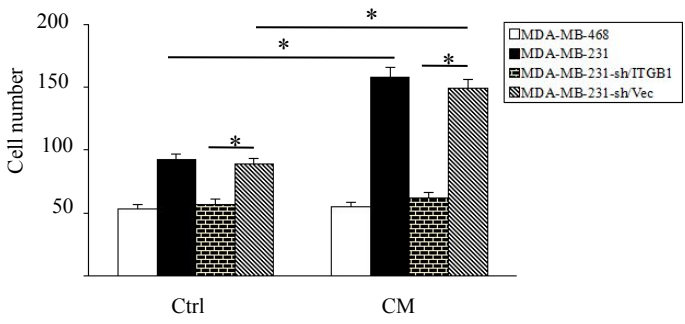

Figure S3

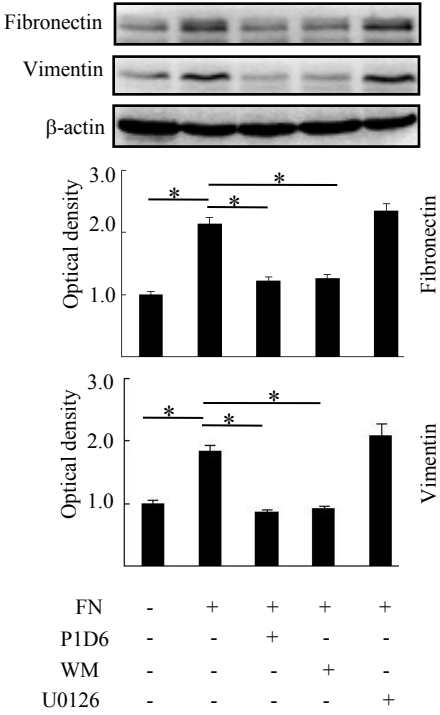

Figure S4

Supplement: Additional file 2: Figure S1. — A second shRNA sequence applied to MCF-7R cells confirms the important role of β1-integrin in tamoxifen resistance of breast cancer. The effect of this shRNA on β1-integrin expression (A), cell growth (B) and cell migration (C) was detected with the same method as previously described. *P < 0.05. Figure S2. GPER mediates the upregulation of β1-integrin induced by tamoxifen in SKBR3 cells. (A) The expression of GPER was knockdown by GPER-specific siRNA transfection in SKBR3 cells. *P < 0.05, versus control. (B) Western blotting analysis was used to test the effect of TAM on the level of β1-integrin treated with or without GPER specific siRNA transfection in SKBR3 cells. *P < 0.05. Figure S3. β1-integrin governs the effect of CAFs on MDA-MB-231 cells migration. (A) β1-integrin expression was determined in cells. (B) Expression of β1-integrin was silenced by lentivirus-mediated shRNA in MDA-MB-231 cells. *P < 0.05, versus control. (C) In vitro Transwell assays were performed using CAF-conditioned medium (CM) or normal medium in MDA-MB-468, MDA-MB-231, MDA-MB-231 cells with silenced β1-integrin (MDA-MB-231-sh/ITGB1) and control cells (MDA-MB-231-sh/Vec). *P < 0.05. Figure S4. Fibronectin induces the EMT process through α5β1-integrin and PI3K/AKT signaling in MCF-7R cells. MCF-7R cells were treated with fibronectin (30 μg/ml; FN) for 48 hours, or pretreated with or without α5β1-integrin inhibitory antibody P1D6 (10 μg/ml), the PI3K inhibitor Wortmannin (10 μM; WM), and the MAPK/ERK inhibitor U0126 (10 μM) prior to FN treatment, and then cell lysates were probed with antibodies against fibronectin and vimentin. *P < 0.05. [file 13058_2015_579_MOESM2_ESM.pdf]
